# Supplementary figures and images for: Muscle contraction type-specific association of acceleration and deceleration performance with rates of force development
Source: PeerJ. 2025 Aug 12;13:e19862. doi: 10.7717/peerj.19862 (PMC12356181; doi:10.7717/peerj.19862)

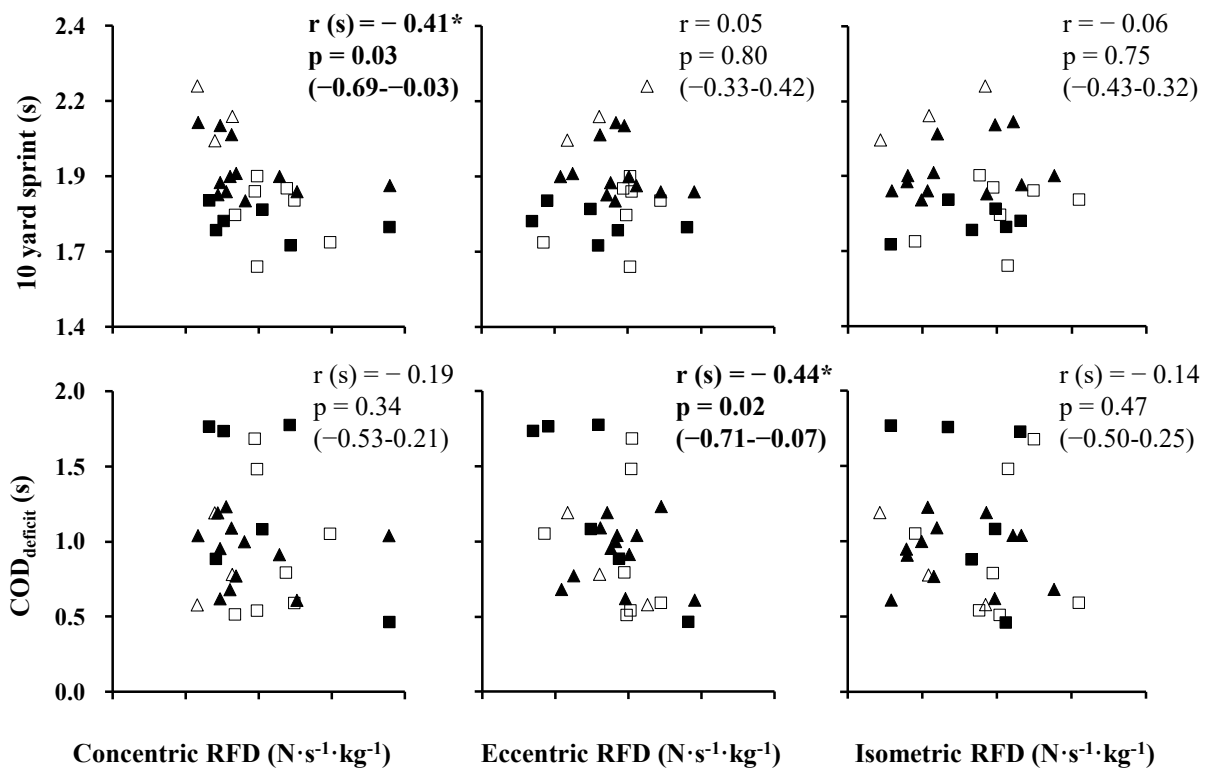

Supplement: Supplemental Information 1 — Filled square: male athletes, empty square: male non-athletes, filled triangle: female athletes, empty triangle: female non-athletes. Pearson product-moment correlation coefficient was written as r. Spearman rank correlation coefficient was written as r (s). 95% confidence intervals are described in parenthese. *p < 0.05. [file peerj-13-19862-s001.pdf]
